# Impact of smell dysfunction of Covid‐19 in older adults on cognition and quality of life

**DOI:** 10.1002/alz.092881

**Published:** 2025-01-03

**Authors:** Latha Velayudhan, James Mitchell, Ellie Pickering, Abbie Palmer, Megan Richards, Zunera Khan, Anne Corbett, Clive G Ballard, Dag Aarsland

**Affiliations:** ^1^ Institute of Psychiatry, Psychology and Neuroscience, King’s College London, London United Kingdom; ^2^ University of Exeter, Exeter United Kingdom; ^3^ University of Exeter, Exeter, Devon United Kingdom

## Abstract

**Background:**

Smell dysfunction has been one of coronavirus disease 2019 (Covid‐19) symptoms. Identification of those with these symptoms are important as olfactory impairment in general has been studied to have increased mortality, poor quality of life, increased incidence of depression and risk for dementia. Smell dysfunction related to Covid‐19 in older adults and its impact is lesser studied. We evaluated impact of covid‐19 infection and smell dysfunction on cognition and quality of life in older adults above 50 years of age.

**Methods:**

A longitudinal online survey, Smell and Taste Function in Older Adults in the Covid‐19 Pandemic (STAP) was added as a sub‐study to the ongoing remote wider study‐ Platform for Research Online to investigate Genetics and Cognition in Ageing (www.protect.org.uk). A separate ethics approval was sought for STAP (Reference LRS‐19/20‐18549). Interested participants of PROTECT study within the UK completed online questionnaire following consent, on a quarterly basis for a year. Information on Covid‐19 symptoms, testing, diagnosis, re‐infections, vaccinations and management was collected as part of the questionnaire in addition to socio‐demographic details, medical and lifestyle history and quality of life.

**Results:**

Of the 3725 participants who took part in the baseline survey since June 2021, 1017 participants completed the follow up survey with the addendum questionnaire on re‐infections, vaccination history and other symptoms including self‐reported cognitive problems. The mean age was 66.65 years (7.87) and 80.5% were women. Of the 437 of the participants who reported having had Covid‐19 infection, 116 participants (26.5%) had partial or total smell loss. Olfactory impairment caused by Covid‐19 was associated with poorer quality of life especially social activities, diet and mood. People with Covid‐19 related smell impairment had significantly higher mental fatigue, attention and concentration problems, difficulties with memory, planning and organising, word and name finding difficulties and making decisions.

**Conclusions:**

Older adults with smell dysfunction related to Covid‐19 infection had poorer quality of life and cognitive abilities affected. Further research is needed not only to understand the longitudinal course of smell impairment related to Covid‐19 and cognition, but also important to devise interventions to support these individuals to improve their well‐being.

